# Mesoporous Platinum Prepared by Electrodeposition for Ultralow Loading Proton Exchange Membrane Fuel Cells

**DOI:** 10.1038/s41598-019-38855-6

**Published:** 2019-03-11

**Authors:** Michael T. Y. Paul, Byron D. Gates

**Affiliations:** Department of Chemistry, Simon Fraser University, 8888 University Drive, Burnaby, B.C. V5A 1S6 Canada

## Abstract

The porosity and utilization of platinum catalysts have a direct impact on their performance within proton exchange membrane fuel cells. It is desirable to identify methods that can prepare these catalysts with the desired features, and that can be widely implemented using existing and industrially scalable techniques. Through the use of electrodeposition processes, fuel cell testing, and electron microscopy analyses before and after fuel cell testing, we report the preparation and performance of mesoporous platinum catalysts for proton exchange membrane fuel cells. We found that these mesoporous platinum catalysts can be prepared in sufficient quantities through techniques that also enable their direct incorporation into membrane electrode assemblies. We also determined that the mesoporous catalysts achieved a high porosity, which was retained after assembly and utilization within fuel cells. In addition, these mesoporous platinum catalysts exhibited an improved platinum mass specific power over catalysts prepared from commercially available platinum nanocatalysts.

## Introduction

Platinum (Pt) is a commonly used catalyst for proton exchange membrane fuel cells (PEMFCs) due to its relatively high catalytic activity, selectivity for the oxygen reduction reaction (ORR), and resistivity to chemical and electrochemical degradation under fuel cell operating conditions^1,2^. To increase the competitiveness of PEMFCs in contrast to internal combustion engines (ICEs) for automotive applications, the mass of Pt used in PEMFCs must be reduced to lower the cost of these systems^3,4^. Researchers have proposed and demonstrated the use of electrodeposition as a superior method for creating catalysts with a higher Pt utilization than the Pt nanoparticle-based powdered catalysts that are widely adopted by current PEMFC manufacturing techniques^5–7^. To the best of our knowledge, this study describes the first demonstration of a potentially commercially viable PEMFC catalyst with an ultra-low loading of Pt and a high degree of mesoporosity as prepared by electrodeposition. The prepared material was analyzed by both *ex-situ* electrochemical and *in-situ* fuel cell techniques. Most notably, membrane electrode assemblies prepared with electrodeposited mesoporous Pt cathode catalyst layers exhibited approximately twice the mass specific power density than conventional Pt nanoparticle-based catalyst. The results of this study demonstrated that the electrodeposited mesoporous catalyst also had a higher utilization of Pt. We anticipate that other platinum group catalysts could be prepared by the electrodeposition technique demonstrated here for use in other electrochemical systems to reduce system costs and improve catalytic efficiencies.

Among the alternative power generation systems, PEMFCs have been an attractive candidate for use in automotive applications due to their high energy density and lower operating temperatures when compared to lithium ion batteries and ICEs, respectively^8–11^. However, the cost of Pt used in both the anode and cathode of PEMFCs can potentially hinder the widespread use of these systems. For example, an increased production of PEMFCs will likely increase the price of Pt because it is a rare earth metal that is at least 1 million times less abundant than iron in the earth’s crust^12,13^. Advances have been made to lower these costs by preparing catalysts using Pt alloys that incorporate more abundant metals, but Pt is still the optimal material for the cathode catalyst in PEMFCs after taking into account both the catalytic selectivity for the ORR and the electrochemical stability of the material^14–18^. Current manufacturing practices aim to increase Pt mass activity and reduce system costs by utilizing Pt nanoparticles (NPs) that maximize the surface area to volume ratios of these catalysts^3,19,20^. During catalyst preparation, these Pt NPs are mixed with support materials that consist of conductive carbon NPs and a non-electrically conductive perfluorosulfonate containing polymer (i.e., a proton conductive ionomer)^12^. The carbon particles and ionomer conduct electrons and protons, respectively, to the Pt surfaces for the catalysis of the ORR. The processing of these catalyst and catalyst support materials can, however, result in agglomeration of the carbon particles that can impede the infiltration of oxygen gas, and the ionomer can coat the conductive particles and insulate them from the conductive pathways within the catalyst layers (CLs)^3,21^. A large amount (ranging from 30 to 80%) of the Pt NPs incorporated into the powder processed, particle-based CLs can be inactive due to the formation of these agglomerates^22–24^. Many investigations have implemented specific engineering controls, such as the ultrasonic dispersion of carbon particles and ionomer for creating catalyst layer inks with reduced aggregation and increased utilization of Pt^25^. To further improve Pt utilization, we believe a fundamentally different approach is necessary for the preparation of CLs for PEMFCs.

Theoretically, Pt nanocatalysts prepared by electrodeposition are superior to those prepared by processing of particle-based inks. Electrodeposition techniques deposit the Pt materials onto regions with sufficient ionic and electrical conductivity^26–30^. The benefits of preparing custom built catalyst materials by electrodeposition was demonstrated in a recent study through the formation of porous Pt on planar glassy carbon substrates created by a pulsed electrodeposition technique^5^. The resulting bicontinuous structure of Pt and void spaces with dimensions <10 nm are more commonly achieved through the selective removal of lesser noble metals from Pt alloys^4,31–33^. These electrodeposited porous Pt structures were determined to have relatively moderate material porosities (25%) and a low overall surface coverage (1.6%)^5^. They demonstrated an ORR mass activity that was at least 9 fold higher than reference catalysts prepared with Pt NPs^5^. These materials were, however, only evaluated through small scale *ex-situ* tests in a solution based electrochemical cell.

In this study, we demonstrated an improved electrodeposition technique that produces mesoporous Pt with relatively high porosities (~60%) and a high surface coverage (>95%). The mesoporous Pt electrodes were prepared using a surfactant assisted process. Characterization of the mesoporous Pt included the use of electron microscopy techniques, such as scanning and transmission electron microscopy (TEM), high resolution electron microscopy, TEM tomography, and energy dispersive X-ray spectroscopy. Further elemental analyses were performed by inductively coupled plasma mass spectrometry (ICP-MS) and X-ray fluorescence (XRF) spectroscopy (e.g., performed on samples both before and after fuel cell testing). Electrochemical tests on the mesoporous platinum were performed in a three electrode electrochemical setup using a rotating disk working electrode, as well as within an assembled fuel cell with a 5 cm^2^ active working area.

## Results and Discussion

**Figure 1:**
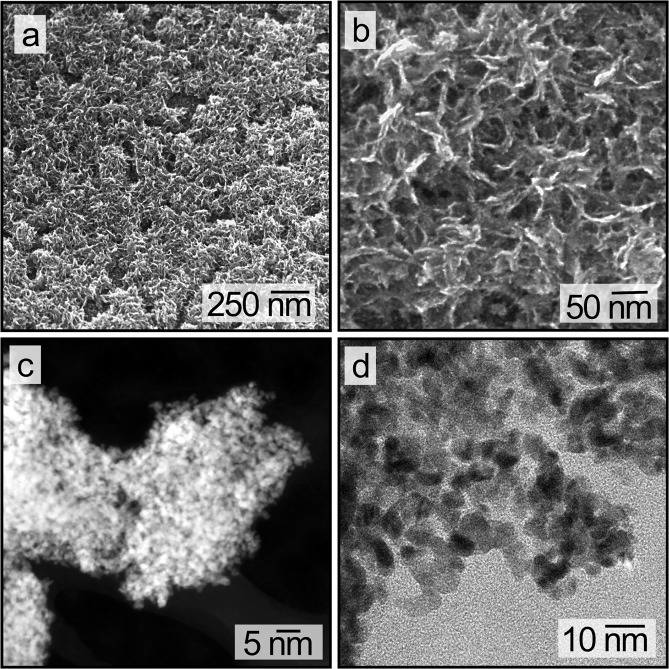
Electron microscopy analyses of mesoporous Pt prepared by electrodeposition. (**a**) Scanning electron microscopy (SEM) image of a surface covered with mesoporous Pt, which was created by electrodeposition for 10 min at 5 mA/cm^2^. (**b**) A higher magnification SEM image of the sample in (**a**). (**c**) Scanning transmission electron microscopy (STEM) analysis using a high angle annular darkfield (HAADF) detector for a section of the mesoporous Pt, and (**d**) a high resolution TEM (HRTEM) analysis of the same sample.

The electrodeposition process was first performed on planar Pt substrates to determine the optimal surfactant system to assist in creating highly porous Pt nanostructures. A range of surfactants, such as polyethylene glycol (PEG), polyethylene glycol hexadecylether (Brij-30), ethylenediaminetetraacetic acid (EDTA), and hexadecyltrimethylammonium bromide (CTAB) were individually evaluated as additives (1.0% v/v) in the solutions of Pt salt used for the electrodeposition process (Table S1). A constant current technique was used for the electrodeposition process to ensure that the production of the mesoporous Pt catalysts could be easily achieved with commonly available industrial equipment. The Pt electrodeposited in the presence Triton X-100 exhibited the highest surface coverage (>95%) and the formation of needle-like nanostructures (Fig. 1a,b), while the other surfactants yielded poorly covered surfaces of feature-less films (Fig. S1). The Triton X-100 molecule has a distinct hydrophobic structure (a 4-tert-octylphenol group) that is covalently linked to a PEG sidechain. The Triton X-100 molecules can exhibit both metal to surfactant interactions (e.g., charge-transfer between the metal surfaces and oxygen atoms within the molecular structure of Triton X-100) and intra-surfactant interactions (e.g., π-stacking of phenol groups and van der Waals forces between alkyl chains) that result in the formation of hydrophobic pockets in solution that are inaccessible to metal ions^34^. The combination of these discrete structures and the relatively high current densities used for Pt electrodeposition (i.e., resulting in a rapid depletion of local concentrations of metal ions) can promote the formation of anisotropic Pt nanostructures^27,35^.

**Figure 2:**
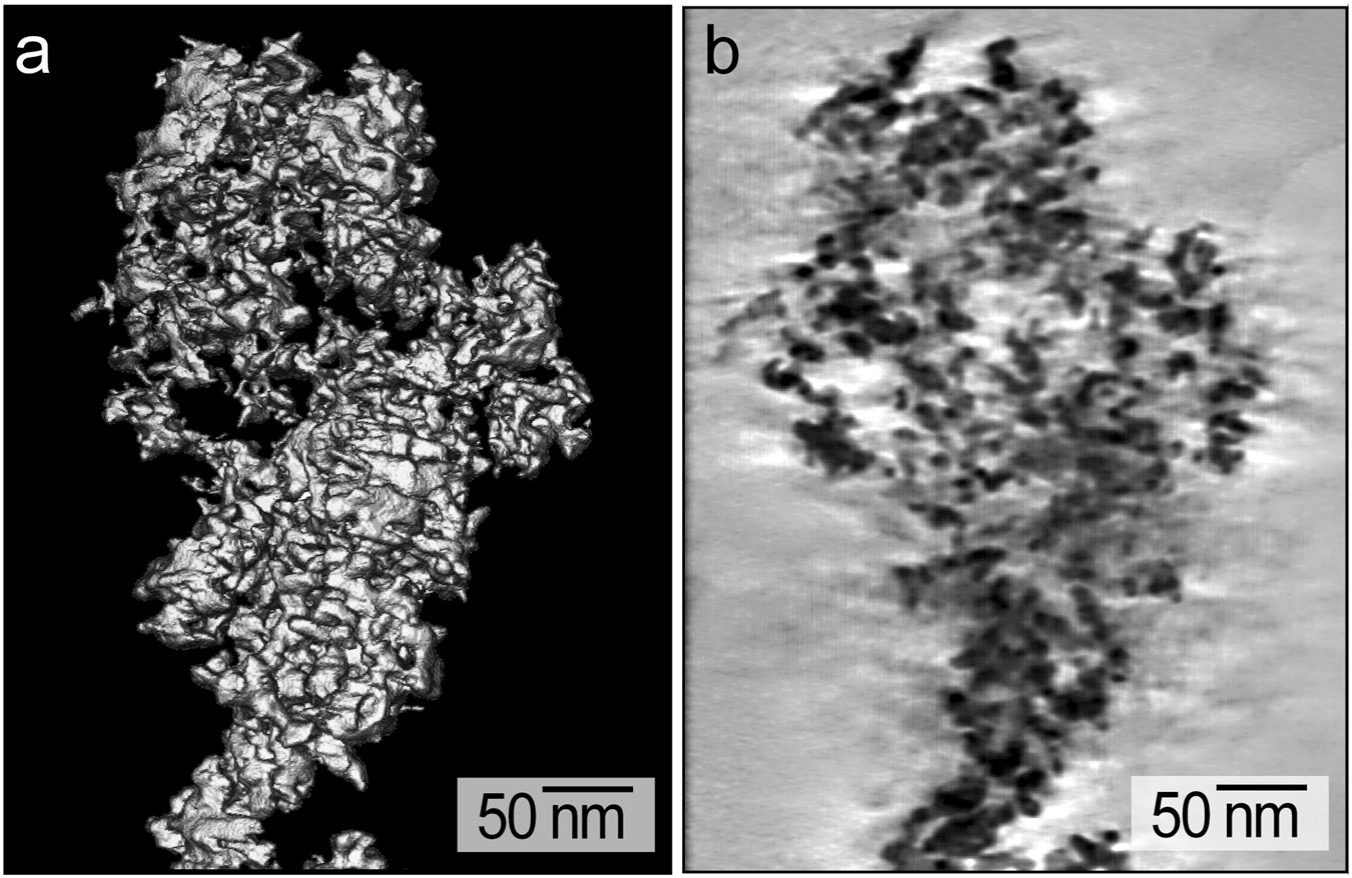
Analysis of the mesoporous Pt obtained by tomographic TEM techniques: (**a**) a 3D rendered image obtained from reconstruction of the tomography results; and (**b**) A reconstructed TEM orthoslice in the XY plane of the mesoporous Pt structure in (**a**).

The porous structure of the electrodeposited Pt prepared in the presence of Triton X-100 was visualized using high resolution TEM techniques (Fig. 1c,d). These TEM analyses indicated that the electrodeposited Pt contained a mesoporous structure of bicontinuous void spaces (with minimum diameters of ~2 nm) and Pt protrusions (with maximum diameters of ~3 nm). The high-resolution TEM analyses of the mesoporous Pt indicated the presence of predominantly (111) planes with an d-spacing of 0.23 nm that is similar to the properties of spherical Pt NPs (Fig. S2)^27^. The average dimensions of the Pt protrusions within these mesoporous structures is similar to the dimensions of Pt NPs commonly used to prepare CLs in PEMFCs^3,21,36^. Three dimensional representations of the mesoporous Pt were prepared using data obtained from TEM tomographic analyses (Fig. 2a). By taking cross-sectional slices from the center of the 3D datasets, the porosity of the mesoporous Pt was determined to be 59 ± 2% (Fig. 2b). This porosity is about 2 to 4 times higher than the porosities reported for nanoporous Pt created by other electrodeposition techniques (e.g., porosities up to 30%, but with average values of 15%)^35,37^. Theoretical studies suggest that cathode CLs containing a porosity of 40 to 50% should exhibit ideal conditions for the mass transport of reagents and products as necessary to optimize the performance of PEMFCs^38–40^. This mesoporous Pt prepared by surfactant assisted electrodeposition was, therefore, a promising material for evaluation of its electrochemical properties, including an assessment of its performance within PEMFCs.

**Figure 3:**
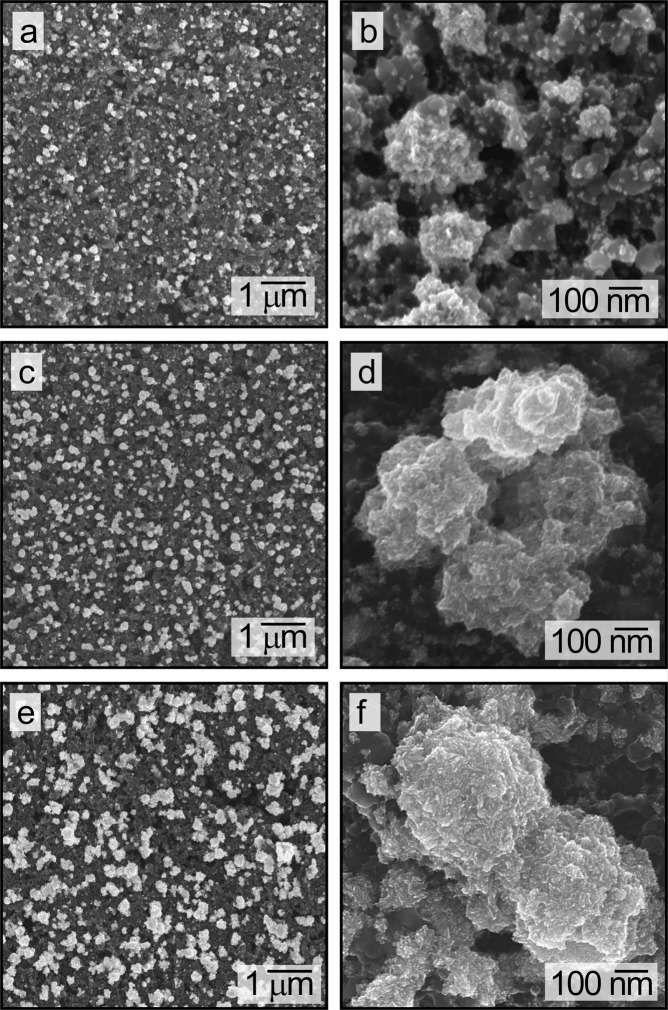
A series of SEM images of mesoporous Pt prepared by electrodeposition onto films containing a mixture of carbon particles and ionomer. The mesoporous Pt were prepared by electrodeposition for: (**a**,**b**) 30 s; (**c**,**d**) 3 min; and (**e**,**f**) 10 min.

To create mesoporous Pt that can be easily incorporated into PEMFCs, the electrodeposition process in the presence of Triton X-100 was carried out on relatively large glassy carbon electrodes (with a working area of ~18 cm^2^) coated with a film of Vulcan XC-72 carbon particles and Nafion^®^ DE2020 ionomer (50% of total carbon weight). Morphology of the electrodeposited Pt, supported on the film of C particles and ionomer, was confirmed by TEM analyses to retain their mesoporous structure (Fig. S3). A series of samples were prepared by electrodeposition of Pt for 30 s, 3 min, and 10 min on the films of C particles and ionomer (Fig. 3). The progressive increase in mass loading of Pt with a longer duration of electrodeposition was investigated as an initial assessment of achieving an optimal loading of mesoporous Pt for use in PEMFCs. This series of C and ionomer supported Pt structures were analyzed and compared for their ORR activity.

**Figure 4:**
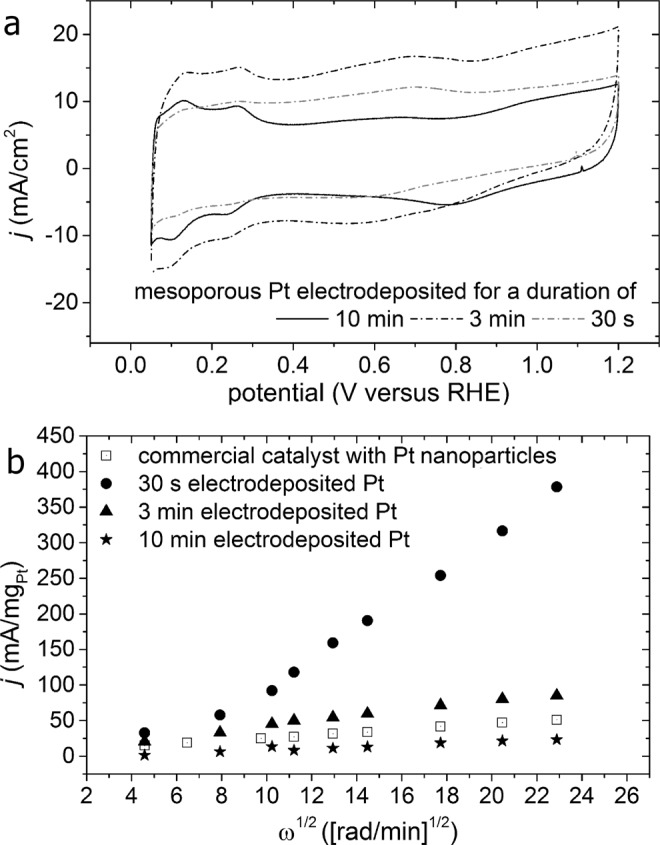
Electrochemical analyses of mesoporous Pt supported on carbon and ionomer films. The mesoporous Pt was prepared by electrodeposition for 30 s, 3 min, or 10 min. (**a**) Cyclic voltammetry (CV) profiles obtained at a scan rate of 100 mV/s while immersing the mesoporous Pt samples in a solution of 0.5 M H_2_SO_4_. (**b**) A Levich plot of the mass activity for each type of mesoporous Pt to the oxygen reduction reaction (ORR) obtained at different electrode rotational speeds. Results are also included for a catalyst ink prepared using commercially available Pt nanoparticles (NPs).

The mesoporous Pt were assessed for the ORR by loading each of these glassy carbon supported layers into a rotating disc electrode setup. These substrates were used as the working electrode in a typical three electrode electrochemical system. The properties of each electrode for the ORR were compared to those of a commercially available catalyst (TEC10V50E, Tanaka Kikinzoku Kogyo, Japan) prepared with a Pt NP loading of 0.2 mg_Pt_/cm^2^ and a CL thickness of ~10 µm. These reference CLs were prepared from a commercially available material of C particles coated with Pt NPs (TEC10V50E, Tanaka Kikinzoku Kogyo, Japan) with an identical amount of ionomer to that used in the samples prepared by Pt electrodeposition. The cyclic voltammetry (CV) profiles of the electrodeposited Pt had well defined peaks associated with the hydrogen desorption and adsorption processes on the Pt (111) and Pt (100) surfaces at ~0.1 and 0.3 V [versus a reversible hydrogen electrode (or RHE)], respectively^41,42^. These features in the CV profiles were more distinct for the mesoporous Pt samples than for the standard catalysts based on Pt NPs (Figs 4 and S4)^43,44^. The CV results suggest that the surfaces of the electrodeposited Pt had fewer interactions with the CL materials. For example, electrolyte access to the Pt surfaces could be impeded by carbon NPs and/or ionomer.

Each type of electrode, including the sample prepared from commercially available materials, was assessed for its efficiency towards the ORR through a series of linear scan voltammetry (LSV) experiments. After electrochemically conditioning the electrodes with a series of CV scans, the electrolyte—initially purged with N_2_—was saturated with O_2_ (99.998%) over a period of 20 min. The ORR efficiencies of the mesoporous Pt prepared by electrodeposition for either 30 s or 3 min each exhibited at least a 2 fold increase in mass activity in comparison to the Pt NP based catalysts. These two types of mesoporous Pt samples maintained a relatively high activity at all rotational speeds of the electrodes in contrast to that of the standard catalyst prepared from a mixture of Pt NPs, C particles, and ionomer (Fig. 4b). In contrast, the sample prepared by electrodeposition of Pt for 10 min exhibited a lower overall efficiency than this standard catalyst under the conditions used for these electrochemical tests. This result suggests that the materials prepared by a longer duration of Pt electrodeposition did not have an equivalent porosity to the other mesoporous materials, or that they exhibited a decrease in the efficiency of electrolyte transport to their electrochemically active surfaces. Either outcome could result from restructuring of the samples during the procedures used for their electrochemical conditioning. A further analysis of these conditioned materials was warranted to assess the probable cause for this apparent decrease in electrochemical activity including an assessment of their mass specific surface area.

The electrodeposited Pt samples were assessed for their electrochemically active surface area (*A*_ecsa_) and their electrochemical stability following a series of electrochemical tests. A series of XRF measurements were obtained at the beginning of test (BOT) and end of test (EOT) time points (Fig. S5). The series of electrochemical tests performed for each sample included 1500 complete CV scans obtained at 100 mV/s, 9 LSV traces obtained at 1 mV/s for a series of different rotational speeds, and 5 complete CV scans obtained at 50 mV/s following each LSV measurement. These tests required at least 12 h to complete. Throughout the duration of these experiments, the electrode of interest was maintained at an applied potential. The XRF measurements obtained at the BOT and the EOT indicated a decrease in Pt content of ~10% (Fig. S5), which may correspond to the dissolution of Pt or a growth in the dimensions of the Pt nanostructures. The growth of the nanoscale features of Pt could increase shielding of the XRF detector from the emitted X-ray signals^45^. Cross-sectional SEM analyses of the electrodeposited Pt was performed at the EOT for samples initially prepared by electrodeposition for 3 min (Fig. S6a,b). The electrodeposited Pt beneath the outermost surfaces of this sample at the EOT still retained relatively small nanostructures of Pt with overall dimensions of ~100 nm. The TEM analysis of these mesoporous Pt at the EOT indicated that their porosity is retained after the electrochemical tests (Fig. S6c). The *A*_ecsa_ per gram of Pt was determined for each sample using the CV profiles obtained at the EOT in combination with their Pt content as determined from ICP-MS analyses at the EOT. Mesoporous Pt samples prepared by electrodeposition for 30 s, 3 min, and 10 min had mass specific surface areas (*A*_ecsa_/g_Pt_) at the EOT of 32.9, 25.6, and 8.5 m^2^/g_Pt_. The Pt catalyst created by electrodeposition over a 10 min period exhibited a significantly lower *A*_ecsa_/g_Pt_ (8.5 m^2^/g_Pt_) in comparison to all of the other samples (≥25.6 m^2^/g_Pt_). This reduction of the *A*_ecsa_/g_Pt_ for the sample prepared at 10 min of electrodeposition further suggested that this sample has a lower activity towards the ORR. The SEM and TEM analyses confirmed both the formation and the retention of mesoporous Pt in these samples after the electrochemical tests. These results suggested that the samples with a relatively higher loading of Pt prepared by 10 min of electrodeposition were hindered by an overall decrease in mass transport of electrolyte to all of the available *A*_ecsa_ during the CV measurements (e.g., at scan rates of ≥50 mV/s). Relatively high scan rates were used in these assessments to mimic conditions that are likely to be experienced within catalyst layers during PEMFC operation that can lead to a decrease in their performance (e.g., flooding with water and/or depletion of dissolved O_2_ within the electrolyte in proximity to the Pt surfaces).

Tomographic analyses by TEM and 3D reconstruction of these datasets for the electrodeposited Pt further supported initial assessments of the porosity and *A*_ecsa_ of these materials. For example, the TEM results indicated that the surface area to weight ratio of the sample prepared at 3 min of electrodeposition is about 22.8 m^2^/g_Pt,_ which is in agreement with the associated electrochemical measurements. The Pt NP based CLs, in contrast, exhibited a mass specific surface area of 68.0 m^2^/g_Pt_. This value is similar to those reported in the literature^3,38^. It was previously determined that *A*_ecsa/_g_Pt_ would need to be ~40 m^2^/g_Pt_ for ultra-low loadings of Pt in the cathode CLs of PEMFCs to be commercially viable for automotive applications^12^. That assessment took into account the relatively high oxygen transport resistances that can arise from the interactions of the ionomer with the Pt NPs. Analyses by high resolution elemental mapping indicated that the C and ionomer supported mesoporous Pt exhibited distinct regions for the fluorine (i.e., ionomer) and Pt signals (Fig. S3). The clear segregation of the F and Pt signals suggests that the mesoporous Pt were only deposited onto the exposed surfaces of C particles that were largely free of ionomer. Although the mesoporous Pt prepared by electrodeposition for 30 s and 3 min achieved only 60 to 80% of the *A*_ecsa_/g_Pt_ target previously reported for ultra-low loadings of Pt in PEMFCs, the surfaces of the mesoporous Pt were largely free of ionomer. These mesoporous structures could form viable catalysts for use in PEMFCs, but their structure (and likely their performance) is distinct from that of commercially available Pt NP catalysts.

The electrochemical tests suggest that there is a higher Pt utilization for these mesoporous Pt samples than for the Pt NP based catalysts. The samples prepared using 30 s and 3 min of Pt deposition in the presence of Triton X-100 exhibited the 1^st^ and 2^nd^ highest mass activities for the ORR, respectively. The commercially available Pt NP catalyst had the highest mass specific *A*_ecsa_, but also had a lower overall mass activity towards the ORR than the mesoporous samples prepared using 30 s and 3 min of Pt deposition (Fig. 4b). It is likely that a portion of the Pt within the sample prepared from the Pt NPs is electrochemically inactive during the ORR^3,21,36^. These reference catalysts prepared from Pt NPs contained a more uniform distribution of Pt throughout the 10-µm thick CLs. Cross-sections at the EOT prepared from the mesoporous sample, originally created by the 3 min electrodeposition process, confirmed the presence of mesoporous Pt at depths of 3 to 5 µm into the film of C and ionomer (Fig. S6). A gradient of Pt coverage was, however, observed with the highest loading of Pt on the outermost surfaces of the C and ionomer layers, which would be in contact with the membrane when assembled into the membrane electrode assembly (MEA). This difference in Pt distribution between the reference CLs and those with mesoporous Pt may improve the Pt utilization, proton conduction, and mass transport characteristics of the mesoporous catalysts within PEMFCs^46^. For example, this CL design could enable a more efficient delivery of reactants (e.g., protons and electrons) to and removal of water from the Pt catalysts^47^. This non-uniform distribution of Pt could result in a better overall performance of the electrodeposited samples for the ORR when compared to the Pt NP based catalyst materials.

**Figure 5:**
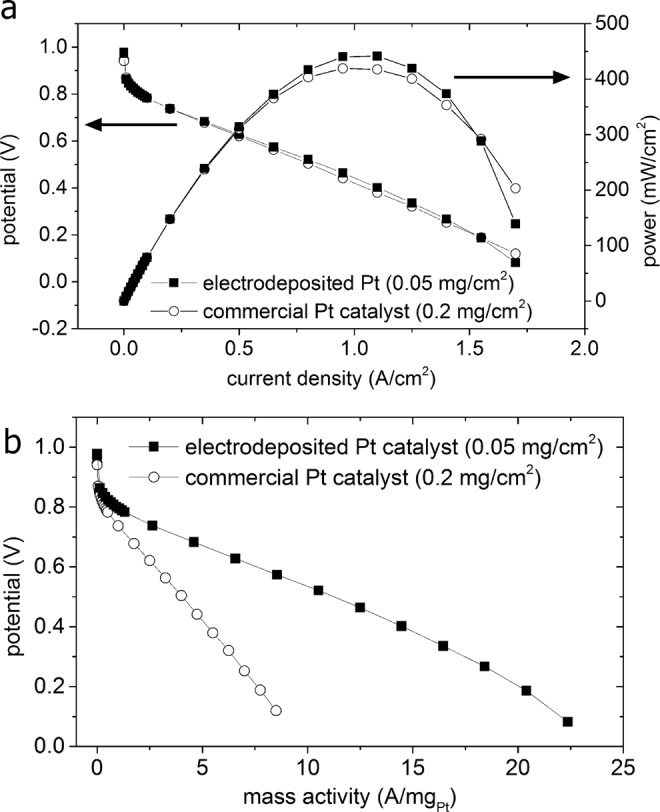
Polarization profiles for proton exchange membrane fuel cells (PEMFCs) prepared with catalysts layers containing either the mesoporous Pt or commercially available Pt NPs. (**a**) Polarization profiles and power curves for the mesoporous Pt or Pt NPs in the cathode catalyst layers after normalization against the active surface area of the fuel cell. (**b**) The same polarization profiles in (**a**) after normalization against the mass of Pt in the cathode CL as determined by X-ray fluorescence spectroscopy (XRF).

Based on the initial electrochemical measurements, mesoporous Pt were prepared on films of C and ionomer through Pt electrodeposition for 3 min and evaluated for their performance in PEMFCs. This electrodeposition time was selected in an attempt to maximize the mass specific surface area of the mesoporous Pt samples under conditions relevant to fuel cell operation, while also maintaining an overall Pt loading that is directly comparable to that used for the Pt NP based catalysts^12,48,49^. Mesoporous samples were prepared using a custom built large area electrochemical cell (Fig. S11). The electrodeposition was performed using a two electrode setup. The CL was coated onto a glassy carbon plate that served as the working electrode with a solution tight glass cylinder serving as the electrolyte reservoir (i.e., defining an electrochemically active, planar area of ~18 cm^2^ on the carbon plate). A graphite rod was used as the counter electrode. Films containing the mesoporous Pt were incorporated into MEAs as the cathode CLs by a process of decal transfer (Fig. S11). Membrane electrode assemblies containing Pt NP based catalysts were prepared as reference materials using two different Pt loadings (i.e., 0.2 and 0.4 mg_Pt_/cm^2^). The PEMFCs were prepared with a 5 cm^2^ active surface area for each CL. The fuel cells were tested under standard operating conditions as outlined by the United States Department of Energy (US DOE)^50^. At least two different MEAs were prepared and tested for each type of the CL (e.g., mesoporous Pt versus Pt NPs) evaluated in these measurements. Typical CV profiles for the different types of MEAs are presented in Fig. S7, which indicated a similar *A*_ecsa_ between the commercial and electrodeposited samples. The average polarization curve for the MEAs prepared with cathode CLs containing the mesoporous Pt exhibited at least a two fold increase in cathode Pt mass activity at 0.7 V (cell voltage) when compared to the MEAs containing cathode CLs prepared from Pt NP based catalysts (Fig. 5a). The mass activities observed for each of the MEAs containing the Pt NP based catalysts were similar despite the two different Pt loadings (i.e., adjusted by tuning the CL thickness). These results suggest that any differences in the thickness of the cathode CLs between the various samples may not play a significant role in their observed fuel cell performance (Fig. S9a). The calculated values of power per mg_Pt_ were also similar for the Pt NP based catalysts prepared with Pt loadings of 0.4 and 0.2 mg/cm^2^ (e.g., 0.49 g_Pt_/kW and 0.45 g_Pt_/kW, respectively) (Fig. S9b). Without correcting for Pt mass activity, the ultra-low loading CLs containing the mesoporous Pt (0.05 mg_Pt_/cm^2^) demonstrated a similar PEMFC performance when compared to the Pt NP based catalysts prepared with a Pt loading of 0.2 mg_Pt_/cm^2^ (Fig. 5b). The MEAs containing mesoporous Pt had an average total Pt mass specific power that was approximately twice the improvement (0.23 g_Pt_/kW) over the PEMFCs containing only CLs prepared from commercial Pt NPs. These results collectively demonstrate that mesoporous Pt created by electrodeposition onto a film of C and ionomer can be directly incorporated into PEMFCs as cathode CLs, and can exhibit higher Pt mass specific activities and powers than observed for Pt NP based catalysts.

In conclusion, it was demonstrated that mesoporous Pt can be created by a one-step electrochemical process under constant current conditions with the addition of appropriate surfactants into the electroplating solution. Evaluation of these mesoporous Pt structures as cathode catalysts in PEMFCs demonstrated that these materials had approximately twice the higher mass specific activity than for Pt NP based catalysts. The results demonstrated that direct electrodeposition of Pt onto C and ionomer, followed by decal transfer to prepare MEAs can provide a superior method for preparing electrocatalysts and improving Pt utilization than powder processing methods. Future analyses of the mesoporous Pt for PEMFCs will include an evaluation of their impact on ionic and oxygen mass transport, stability to extensive corrosion cycles, and incorporation into larger area MEAs (>40 cm^2^) to further verify the suitability of these materials and methods to prepare cathode catalysts for PEMFCs. The results of these initial studies suggest that the electrochemical deposition of electrocatalysts warrants a further investigation by the fuel cell industry as a method to increase Pt utilization and to reduce system costs. The preparation of mesoporous Pt materials could also be applied to other electrochemical systems, such direct methanol and metal-air fuel cells.

## Supplementary information

Supplementary Information
