# Supplementary material for: Mesoporous Platinum Prepared by Electrodeposition for Ultralow Loading Proton Exchange Membrane Fuel Cells
Source: Sci Rep. 2019 Mar 11;9:4161. doi: 10.1038/s41598-019-38855-6 (PMC6411755; doi:10.1038/s41598-019-38855-6)
Supplement: Supplementary file 1 — Supplementary Information [file 41598_2019_38855_MOESM1_ESM.pdf]

Supplementary Information

Mesoporous Platinum Prepared by Electrodeposition for Ultralow Loading Proton Exchange Membrane Fuel Cells

Michael T.Y. Paul and Byron D. Gates*

Department of Chemistry, Simon Fraser University

8888 University Drive, Burnaby, B.C. V5A 1S6, Canada

*E-mail: bgates@sfu.ca

This research was financially supported in part by from the Natural Sciences and Engineering Research Council (NSERC) Discovery Program (Grant No. 1077758), CMC Microsystems (MNT Grant No. 4440), and the Canada Research Chairs Program (B.D. Gates; Grant No. 950-215846). This work made use of 4D LABS (www.4dlabs.ca) and the Centre for Soft Materials shared facilities supported by the Canada Foundation for Innovation (CFI), British Columbia Knowledge Development Fund (BCKDF), Western Economic Diversification Canada, and Simon Fraser University. We also thank Dr. Matt Bilton for assistance in obtaining the series of transmission electron microscopy images at various tilts for the tomography analyses, and Dr. Stefano Rubino for assistance in performing the ICP-MS analyses.

Materials and Methods

Substrate Preparation

Mesoporous Pt catalysts were prepared by electrodeposition onto a variety of substrates to characterize the deposition process. The electrochemical activities of these nanostructures for the oxygen reduction reaction (ORR) were compared to commercial catalysts of Pt nanoparticles. The performance of these catalysts were evaluated through a series of tests in electrochemical rotating disc electrode (RDE) and proton exchange membrane fuel cell (PEMFC) setups.

A planar conductive Pt substrate was initially used to identify appropriate surfactants for use in the electrodeposition process, and to assess the current densities required for the formation of mesoporous Pt. A p-type <001> Si wafer was coated with a 5-nm thick Cr adhesion layer and a 150-nm thick layer of Pt, which was used as a planar substrate for the electrodeposition processes. These Cr and Pt metal films were sequentially deposited by thermal evaporation, without breaking vacuum, using a custom built physical vapor deposition (PVD) system in 4D LABS (Simon Fraser University).

Mesoporous Pt was also deposited onto glassy carbon electrodes that were coated with a layer of Vulcan XC-72 (Cabot Corporation, United States) carbon particles and ionomer (Nafion® DE2020, Dupont, United States) — referred to as the C and ionomer films in the main text. This layer of catalyst free C and ionomer film mimicked was prepared similarly to the reference CCLs. The similarities in CL preparation and layer thickness allowed a fairer comparison of the electrochemical performance of these mesoporous Pt catalysts to conventional CCLs prepared with Pt nanoparticles. The glassy carbon electrodes (5-mm in diameter) were inserted into a ChangeDisk electrode holder (Pine Research Instrumentation; NC, United States) and used as the working electrodes to support the Pt nanostructures during their electrochemical analyses by RDE

techniques. The glassy carbon electrodes were each sequentially polished using a suspension of 300-nm and 50-nm diameter alumina particles (Buehler, IL, United States) to create a mirror-like finish. The polished carbon was coated with an ~10-µm thick layer of Vulcan XC-72 carbon nanoparticles mixed with a 30 wt % loading of ionomer (Nafion® DE2020, Dupont, United States). This layer of carbon particles and ionomer was prepared by spin casting this ink mixture at 500 rpm for 5 min prior to Pt electrodeposition to mimic the structure and composition of conventional CCLs in PEMFCs. Prior to spin coating, the suspension of carbon and ionomer were dispersed in a 3:1 (w/w) mixture of 2-propanol (Anachemia, Canada, ACS grade) and deionized (DI) water to achieve a 0.3 w/w % (solid/liquid) solution. All DI water, used to prepare the necessary solutions and for rinsing of the electrodes, was filtered with a Barnstead DIamond™ deionizing water system with an output of 18 MΩ·cm. An aliquot of 20 mL of the dilute ink solution was placed into a glass vial. The vial containing the ink solution was chilled by placing it into an ice filled container and sonicated using a process of 1 s "on" and 3 s "off" for a total duration of 1 h (and a total of 15 min of sonication during the collective "on" time) using a probe sonicator (Fisher Scientific, United States, Sonic Dismembrator 500). A 12-mm diameter sonication probe was immersed ~1 cm into the solution, and operated at 40 % of the maximum power (the maximum power was ~500 Watts).

Large area glassy carbon electrodes were also coated with the layer of carbon particles and ionomer upon which the electrodeposited mesoporous Pt were prepared and transferred to membrane electrode assemblies (MEAs) for fuel cell testing. To prepare these films containing a layer of mesoporous Pt, a 3-mm thick and 5-cm by 5-cm wide square glassy carbon plate (SPI Supplies, United States) was first spin coated (at 500 rpm for 30 min) with the layer of carbon particles and ionomer, and subsequently loaded into a custom electrodeposition cell (see Figure

S11 for more details). The electrodeposition of mesoporous Pt were performed on planar Pt electrodes, C and ionomer coated glassy carbon RDE electrodes, and C and ionomer coated large area glassy carbon electrodes to determining the optimal electrodeposition conditions, to analyze the electrochemical characteristics of the mesoporous Pt catalyst, and to prepare mesoporous Pt for incorporation into the MEAs for PEMFC analyses.

Electrodeposition of Mesoporous Pt

Planar Pt electrodes, C and ionomer coated glassy carbon RDE electrodes, and C and ionomer coated large area glassy carbon electrodes were each separately used as the working electrode during the electrodeposition of mesoporous Pt. A series of surfactants, such as polyethylene glycol (PEG; average Mn ~400; Sigma Aldrich, United States), ethylenediaminetetraacetic acid (EDTA; Sigma Aldrich, ACS Grade, United States), hexadecyltrimethylammonium bromide (CTAB; ≥96 % [AT]; Sigma Aldrich, United States), and polyoxyethylene (30) cetyl ether (Brij-30; Sigma Aldrich, United States) were each evaluated for their influence on creating mesoporous Pt. Following this initial screening process, a series of mesoporous Pt were prepared by electrodeposition from an aqueous solution of 5 mM H_2PtCl_6 (Sigma Aldrich, ACS Grade, United States), 0.2 M H_2SO_4 (Caledon Laboratories Ltd, ACS Grade, Canada), and 1.0 % (v/v) polyethylene glycol p-(1,1,3,3-tetramethylbutyl)-phenyl ether (Triton™ X-100; Sigma Aldrich, Laboratory Grade, United States). Each of the prepared solutions were stored in the dark at room temperature for at least 24 h prior to use in the electrodeposition processes to ensure all of the respective reagents were in equilibrium with one another. Electrodeposition of Pt onto each of the desired conductive substrates was performed using a SP-150 potentiostat (BioLogic Science Instruments, France). The electrodeposition processes were

performed galvanostatically at 5 mA/cm^2 for 30 s, 3 min, or 10 min with a graphite rod (part #: MPGRR250; Pine Research Instrumentation, NC, United States) as the counter electrode in a two electrode electrochemical setup.

Electron Microscopy Characterization

Electron microscopy techniques, such as scanning electron microscopy (SEM) and transmission electron microscopy (TEM), were used to verify the surface coverage, morphology, and crystalline nature of the electrodeposited Pt. The SEM analyses were performed using an FEI Helios SEM/ focused ion beam (FIB) dual beam system operating at 10 kV. This system was also equipped with an EDAX energy dispersive X-ray spectroscopy (EDS) detector for elemental mapping of the materials. The surface coverage of mesoporous Pt over the layers of C particles and ionomer were calculated from a series of SEM images by using image segmentation with assistance from ImageJ (ImageJ 1.x), an open source software package.[1] This analysis assumed that isolated regions of the underlying planar substrate with a darker (lower) contrast in the SEM images were not covered by the mesoporous Pt and, hence, exhibited a distinct contrast in the production of secondary electrons. The regions containing Pt were brighter, and could be resolved to a higher resolution as observed in the representative SEM images throughout the manuscript and SI file. The regions indicated by the image segmentation by ImageJ were manually checked to ensure proper assessment of the regions of interest. At the end of a series of electrochemical tests the electrode materials were sacrificed for preparing cross-sections. The experimental procedures for these electrochemical tests consisted of the following series of techniques: (i) 1500 complete cyclic voltammetry (CV) scans at 100 mV/s from 0 to 1.2 V [versus a reversible hydrogen electrode (or RHE)]; (ii) linear scan voltammetry (LSV) measurements at 1 mV/s from

1.2 to 0.2 V (versus RHE) at a series of different electrode rotational speeds; and (iii) 5 complete CV scans at 50 mV/s from 0 to 0.8 (versus RHE) after each LSV measurement. These tests required at least 12 h while continuously holding the electrode of interest under an applied potential. Cross-sections of the samples for SEM analysis were obtained by lifting out sections of the catalyst films through the assistance of small sections of adhesive carbon tape (Ted Pella, Inc., CA, United States). For TEM analyses, sections of the electrodeposited samples were physically removed from the Pt or carbon electrodes using a plastic spatula. These sections of each sample were collected into centrifuge tubes containing 50 μL of DI water. A 10 μL portion of each suspension was drop cast onto Formvar/carbon coated 300 mesh copper TEM grids (PELCO®, Ted Pella Inc., United States) for analysis by TEM and EDS techniques. All of the TEM related analyses were performed using an FEI Osiris X-FEG S/TEM operating at 200 kV. The TEM was equipped with a Super-X EDS detection system and a Fischione single tilt holder (advanced tomography holder Model 2020, Fischione Instruments, Inc.; United States). The TEM tomography analyses were prepared from a series of images obtained at tilt angles from +70° to -65° with 3° increments between each image. The series of tomography images were processed by Inspect 3D Express (FEI, Thermo Fisher Scientific, United States) for tilt correction, alignment, and volume reconstruction of the data. The reconstructed volumes were rendered by Amira Version 6.0 (FEI, Thermo Fisher Scientific, United States).

Electrochemical Characterization

A series of electrochemical experiments were performed to determine the electrochemically active surface area (A_{ecsa}) of the samples, to condition the electrodes for the ORR experiments, and to determine the ORR efficiency of each electrode by CV and LSV techniques.

These experiments were performed using a BioLogic Science Instruments SP-150 potentiostat in addition to using an analytical rotating disk electrode (RDE) system (Modulated Speed Rotator, Pine Research Instrumentations, PA, United States). Electrochemical data was collected using EC-Lab data analysis software (V10.18). The as-prepared electrodes were incorporated as the working electrode into a typical three electrode setup using a custom built glass electrochemistry cell with a custom made Pt reversible hydrogen reference electrode, and a Pt gauze counter electrode (100 mesh woven from 0.0762-mm diameter; 99.9 %; Alfa Aesar, United States). All electrochemical experiments were performed in a 0.5 M H_2SO_4 solution that was purged with ultrahigh purity N_2 gas (99.999 %, Praxair, Canada) for at least 30 min prior to the commencement of the experiments. A positive N_2 pressure was maintained in the head space of the electrochemical cell throughout the experiments unless otherwise noted. The CV measurements were performed without rotation at a scan rate of 100 mV/s from 0 to 1.2 V (versus RHE) while at ambient temperatures (measured to be 22.1 ±0.2 °C). Prior to the ORR analyses, a series of CV scans were performed on each electrode to optimize their ORR activities and to remove any adsorbed organic molecules from the Pt surfaces (e.g., from the electrodeposition process or carbon contaminating species from the atmosphere). A total of 1500 complete CV scans were performed to condition the electrodes to maximize the ORR performance of the electrodes and ensure consistency of the ORR performance between different CLs. Prior to the ORR experiments, O_2 gas (99.993 %, Praxair, Canada) was bubbled through the electrolyte for at least 30 min to saturate the solution with dissolved oxygen. The subsequent LSV experiments for the ORR were performed with the same electrochemical set-up, but at a series of electrode rotational speeds and with potentials scanned from 1.2 to 0.2 V (versus RHE) at a scan rate of 1 mV/s. The range of rotational speeds included 200, 600, 1000, 1200, 1600, 2000, 3000, 4000, and 5000 rpm. These parameters were chosen to assess the catalytic

efficiencies of the samples towards the ORR while immersed in the sulfuric acid solutions. Furthermore, the number of electrons involved in the ORR were investigated using a Koutecký-Levich plot (current density versus the inverse square root of the rotational speed in rad/s) generated from the series of RDE experiments performed for each of the electrodes. A representative analysis is presented in Figure S10.

A total of five CV scans at 50 mV/s were performed, from 0 to 0.8 V versus RHE, between each consecutive LSV measurement as part of the ORR analysis to ensure a consistency of the Pt catalysts. The CV profiles were used to assess the A_{ecsa} of the Pt for each of the electrodes (e.g., Figure 4a). The A_{ecsa} of Pt was calculated from the area under the hydrogen desorption peaks assuming that the charge associated with the formation of a monolayer of hydrogen on the Pt surfaces is approximately 210 μC/cm^2.[2–4] The A_{ecsa} per gram of Pt was determined following the series of electrochemical analyses. This series of electrochemical tests, which were performed over a continuous period of at least 12 h, were followed by X-ray fluorescence spectroscopy (XRF) and inductively coupled plasma mass spectrometry (ICP-MS). This order of experiments was adopted due to the destructive nature of the ICP-MS analyses.

Preparation of Catalyst Coated Membranes

A series of MEAs were prepared and tested to characterize the electrodeposited mesoporous Pt for its performance in PEMFCs. To compare these materials with conventional cathode catalysts for PEMFCs, MEAs were prepared using cathode catalysts containing a mixture of commercially available Pt nanoparticles coated C particles (TEC10V50E, Tanaka Kikinzoku Kogyo, Japan), and ionomer (Nafion® DE2020). A series of MEAs were also prepared using mesoporous Pt electrodeposited onto films of carbon particles (Vulcan® XC-72) mixed with

ionomer. The standard anode and cathode catalyst inks were prepared according to the same procedures outlined in the section providing details on the preparations of the substrates. The same procedures outlined therein for preparing the solutions of C particles and ionomer were also used to create supporting materials for the electrodeposition of mesoporous Pt.

These anode and cathode catalysts were hot-bonded with a proton exchange membrane to form catalyst coated membranes (CCMs) by a process of decal transfer.[5] These catalyst materials were transferred from the substrate used in the electrodeposition process and/or from catalyst coated polymer decals to the membranes through a process of hot-bonding. The standard cathode and anode catalysts were prepared for decal transfer by coating thin films of ethylene tetrafluoroethylene [ETFE; poly(ethene-co-tetrafluoroethene)] with the ink solutions using a generic Mayer bar coater operated at a roller speed of 8 m/min. The catalyst films were dried on a heated vacuum stage at 50 °C for approximately 10 min. The Pt loading and thickness of the cathode and anode catalyst films were controlled using Mayer bar rollers of different sizes. The anode loadings were consistently prepared at 0.1 mg_{Pt}/cm^2. The thicknesses of the cathodes containing Pt NP-based catalyst were varied to prepare Pt loadings of 0.2 mg_{Pt}/cm^2 and 0.4 mg_{Pt}/cm^2. The CCMs were prepared with mesoporous Pt supported on a film of carbon particles and ionomer (itself supported on a glassy carbon plate) as the cathode layer and the Pt NP-based catalyst coated on an ETFE decal as the anode layer. These catalyst layers were both decal transferred at the same time onto the Nafion® membrane at 20 bar of pressure and 150 °C for 5 min with a use of a Carver® 25 ton hydraulic unit (Carver Inc., United States). The reference samples were each prepared by the same hot-bonding conditions for both the cathode catalysts and anode catalysts, which were prepared using the Pt NP containing ink coated onto ETFE decals. The different components used in the hot-bonding process are depicted in Figure S11. A sheet of

0.05-mm thick Teflon® was placed on top of the layered CCMs, followed by a sheet of 2-mm thick polyurethane rubber prior to hot bonding to ensure an even distribution of the bonding pressure. After 5 min of bonding, the layered CCMs were immediately removed from the Teflon® sheet and allowed to cool to room temperature for at least 10 min. The ETFE and glassy carbon plate were gently removed by hand after the CCM assembly was sufficiently cooled. The MEAs were prepared by hot bonding the CCMs with of macroporous layer (MPL) coated gas diffusion layer (GDL) (Sigracet® gas diffusion layers, SGL Group, Germany) and adding a piece of G10 gasket on either side of the CCM. The hot bonding procedure was identical to the one outlined above for the preparation of the CCMs except that the bonding time was reduced from 5 min to 2 min. Additional Mylar® gaskets (DuPont Teijin Films, UK) were used to adjust the overall thickness of the gaskets as necessary to induce a 25 % compression of the MPL/GDL materials when assembled for testing in the fuel cell.

Fuel Cell Testing

The MEAs were analyzed in a single stack test setup for PEMFCs to compare the performance of the mesoporous Pt serving as CCLs versus CCLs prepared from inks containing Pt NP-based catalysts. Fuel cell testing was performed using equipment provided by the Centre for Soft Materials in 4D LABS at SFU. These tests used a 100 W, Teledyne Medusa /Scribner system (Teledyne Technologies Inc., United States). The single stack fuel cells were assembled using graphite plates with parallel flow-field landings. The active area of the MEA exposed in each of the fuel cells was 5 cm^2. Maximum backpressures were maintained at 5 psi for both the anode and cathode. Gas flow rates for the cathodes and anodes were set at 1 L/min (maximum of the system). Electrochemical experiments on the MEAs, such as CV conditioning (200 consecutive

cycles) and the A_{ecsa} determination, were performed using a VersaSTAT 4 potentiostat (Princeton Applied Research, United States). These experiments were performed with a scan rate of 100 mV/s with a scan range from 0 to 1.2 V (verses anode). The cell conditions were maintained at the standard conditions outlined by the United States Department of Energy (US DOE) [cell temperatures of 80 °C with H_2 (99.95 %, Praxair Canada) and N_2 (99.999 %, Praxair Canada) gases supplied to the anode and cathode, respectively, at 100 % relative humidity (RH)].[6,7] The MEAs were further tested under similar conditions to these electrochemical experiments, except O_2 (99.998 %, Praxair Canada) was supplied instead of N_2 (99.999 %, Praxair Canada) at the cathode. The LSV polarizations were performed under potentiostatic conditions from 0.95 to 0.1 V while stepped at 0.05 V increments with a 10 min dwell time at each voltage set-point. A minimum of 12 LSV experiments were performed to condition the MEAs for obtaining stable fuel cell polarization curves. An example of the LSV conditioning profiles are shown in Figure S8. The MEAs were deemed to be stable when at least three consecutive LSV experiments exhibited an overall deviation of < 0.5 mA/cm^2. After the LSV measurements had reached stabilization, 3 additional LSV experiments (100 mV/s; 0 to 1.2 V versus RHE) were performed, the results averaged, and analyzed to assess the fuel cell performance for each of the MEAs.

Analysis of Elemental Composition

Quantitative elemental analysis techniques were used to compare the Pt loadings of the samples. The Pt loading of the samples were initially verified by XRF spectroscopy (Thermo Scientific, Niton XL3t, United States; operating at 50 kV), which was calibrated with a series of Pt thin film standards (ranging from 10 μg_{Pt}/cm^2 to 400 μg_{Pt}/cm^2 in 50 μg_{Pt}/cm^2 increments) made by nanoXRF (UHV Technologies, Inc., United States). For a more accurate determination of the

Pt loadings, the Pt samples were digested at 80 °C in a solution of aqua regia and analyzed by ICP-MS. The aqua regia solutions were prepared as 3:1 (v/v) solutions of hydrochloric acid (36.5 to 38.0 % in water; Anachemia, Canada) and nitric acid (68 to 70 % in water; Anachemia, Canada). *CAUTION: Aqua regia solutions are extremely corrosive. These solutions should be handled with extreme care.* The ICP-MS measurements were performed using an Agilent 7700x quadrupole system maintained by the Department of Chemistry at the University of British Columbia.

Table S1. Physical properties of some surfactants utilized in evaluating the preparation of mesoporous Pt by electrodeposition techniques (data obtained from Sigma Aldrich, Canada).

molecule	average molecular weight (g/mol)	density (g/cm^3)	type of surfactant
CTAB	364	0.39	ionic
PEG-400	400	1.13	non-ionic
Triton™ X-100	625	1.07	non-ionic
Brij-30	363	0.95	non-ionic
EDTA	363	0.86	ionic

Abbreviations:

CTAB = hexadecyltrimethylammonium bromide

PEG 400 = polyethylene (400) glycol

Triton™ X-100 = polyethylene glycol tert-octylphenyl ether

Brij-30 = polyoxyethylene (30) cetyl ether

EDTA = ethylenediaminetetraacetic acid

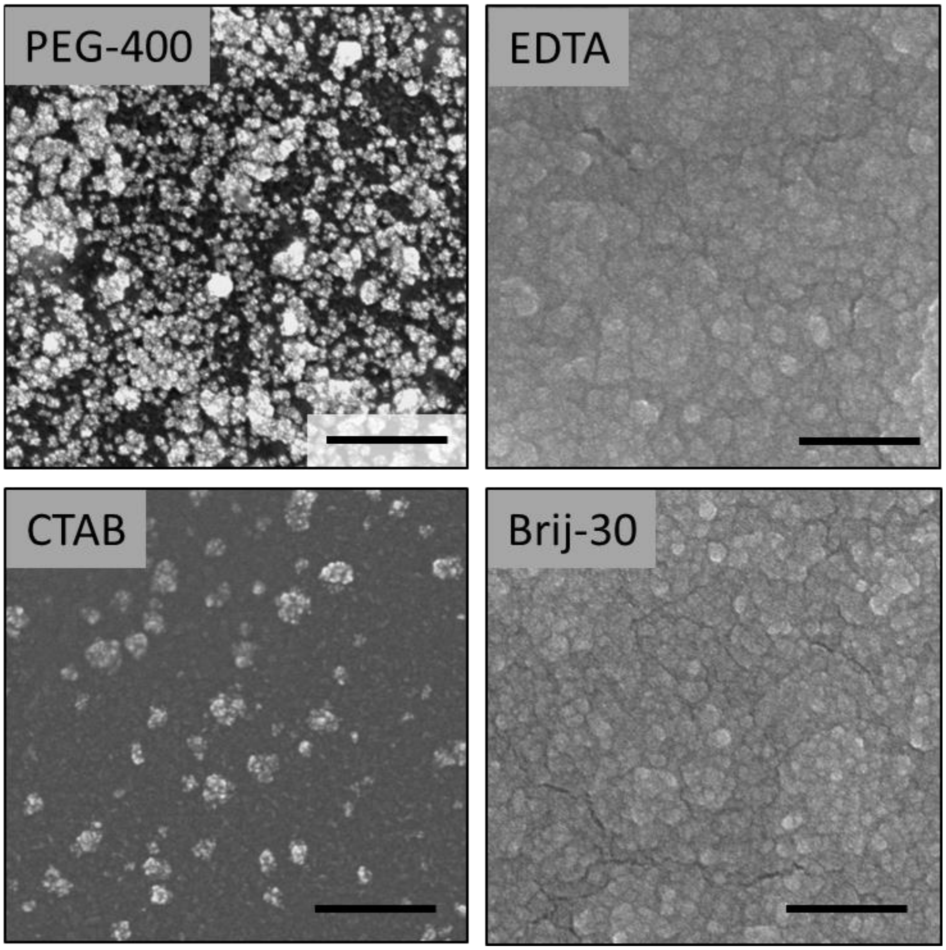

Figure S1. Scanning electron microscopy (SEM) images of Pt nanostructures created by electrodeposition from a solution of 0.5 M H_2SO_4 and 5 mM H_2PtCl_6, in the presence of different surfactants at concentrations of 1.0 % (v/v). These structures were electrodeposited onto electrodes prepared from Pt thin films on polished Si wafers. The surfactant used to facilitate the formation of the electrodeposited Pt structures are indicated on each of the images. The scale bars in each of the images is 200 nm in length.

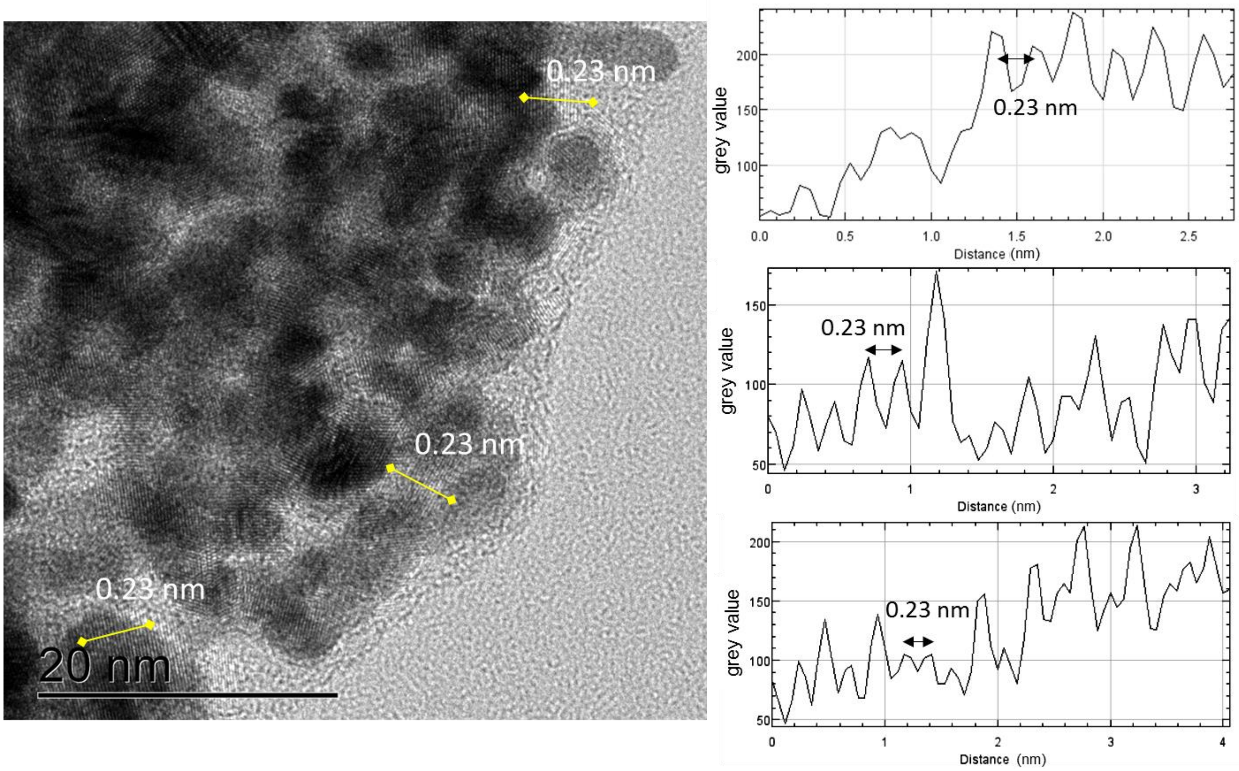

Figure S2. High resolution transmission electron microscopy (HRTEM) analyses of mesoporous Pt depicting lattice fringes that correspond to the spacing of Pt (111) crystal planes.[8]

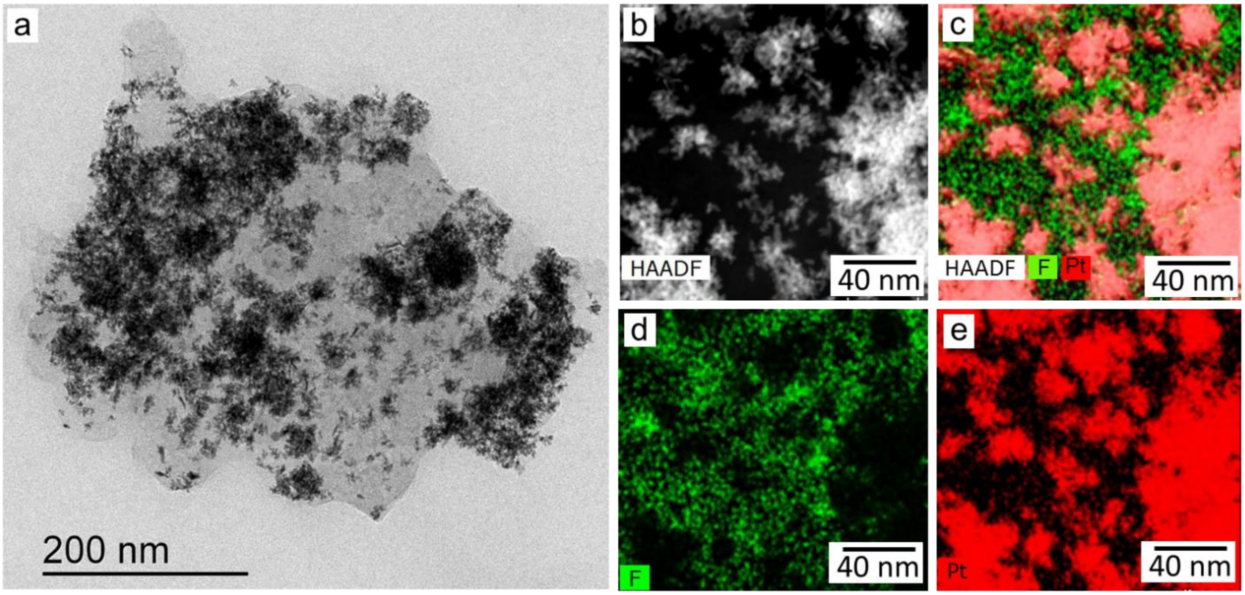

Figure S3. Electron microscopy images of mesoporous Pt that was prepared by electrodeposition on a substrate of carbon and ionomer: (a) transmission electron microscopy (TEM) image of a section of the carbon and ionomer supported mesoporous Pt; (b) high angle annular darkfield (HAADF) image from a section of the sample in (a); (c) overlaid energy dispersive X-ray spectroscopy (EDS) elemental maps corresponding to F and Pt; (d) EDS elemental map for F; and (e) EDS elemental map for Pt corresponding to the sample in (b).

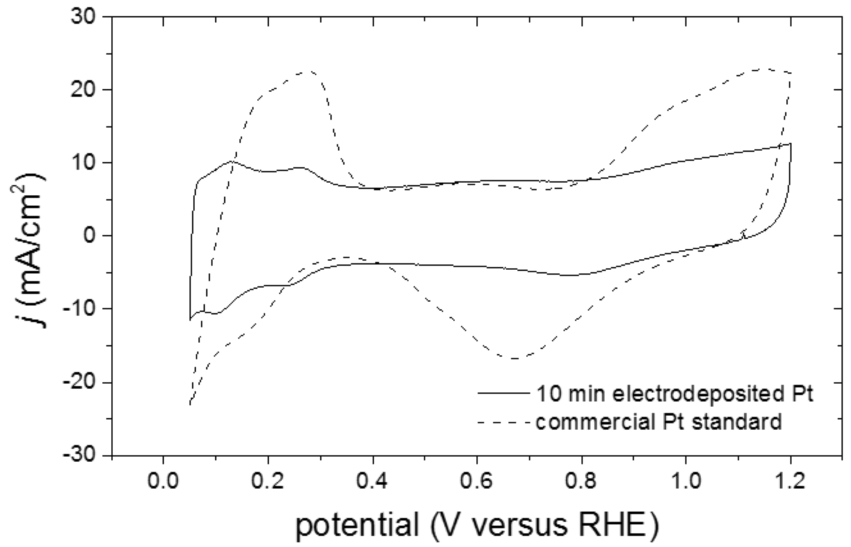

Figure S4. Cyclic voltammetry (CV) profiles for a film prepared from a standard commercial Pt nanoparticle (NP) based catalyst (containing a mixture of Pt nanoparticles, C particles, and ionomer), and for a sample of mesoporous Pt prepared by 10 min of electrodeposition on top of a film prepared from a mixture of carbon particles and ionomer. These samples were each analyzed in a degassed solution of 0.5 M H_2SO_4 at a scan rate of 100 mV/s.

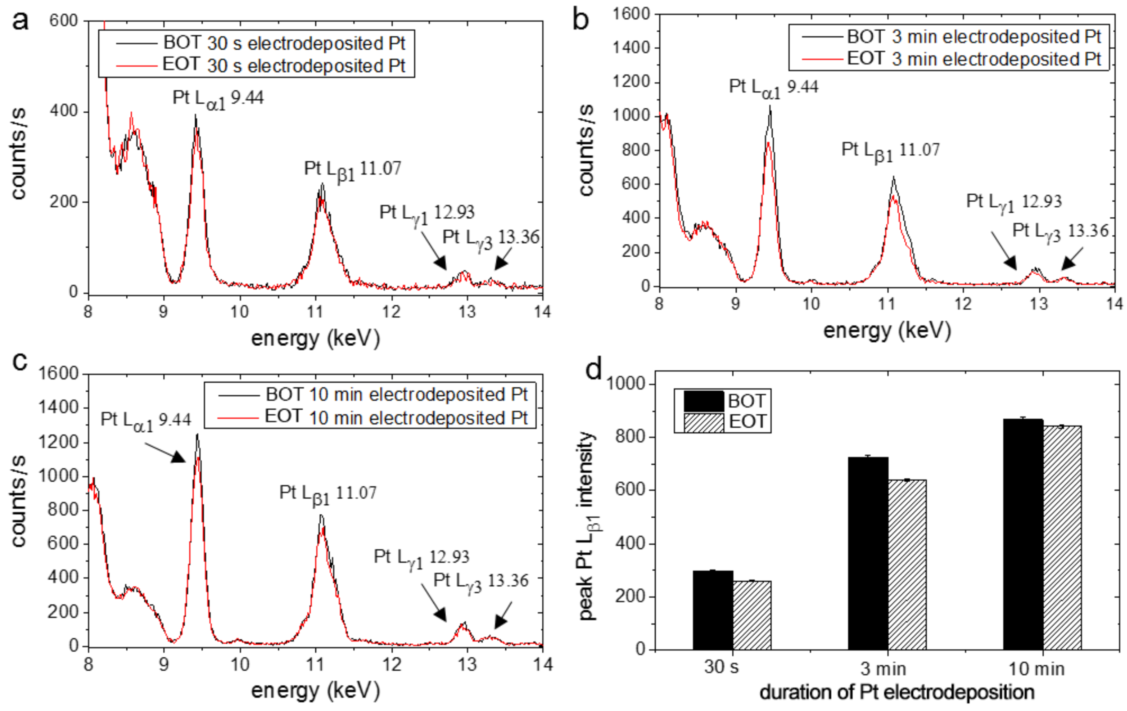

Figure S5. Assessment of the durability of electrodeposited mesoporous Pt before and after electrochemical testing. (a-c) Results from the X-ray fluorescence (XRF) spectroscopy analyses of the mesoporous Pt samples before and after the series of electrochemical tests performed for each sample over a period of at least 12 h. (d) The average peak Pt $L_{\beta 1}$ intensity for each of the samples shown in (a-c) with error bars corresponding to one standard deviation from the calculated mean values. Abbreviations: BOT: beginning of test; and EOT: end of test.

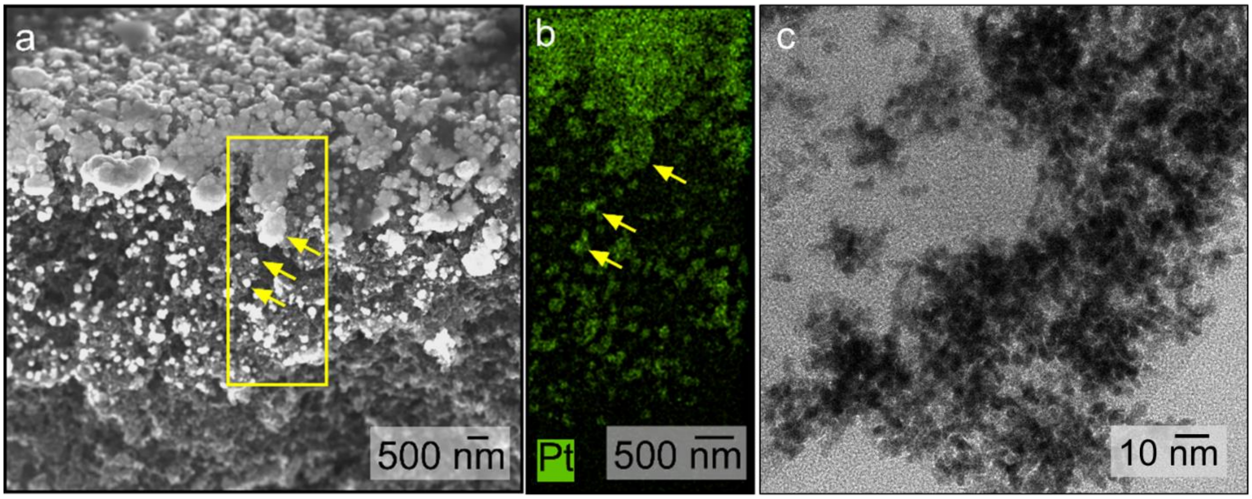

Figure S6. Scanning electron microscopy image of (a) a cross-section obtained at the end of the electrochemistry experiments for mesoporous Pt supported on a layer of carbon particles and ionomer. (b) An EDS map corresponding to the region of interest outlined with a yellow box in (a) with arrows pointing to examples of corresponding features in the two images. (c) A TEM analysis for a sample of Pt, carbon particles, and ionomer obtained from the materials in (a). This TEM study confirmed the presence of the mesoporous Pt structure after the series of electrochemical tests.

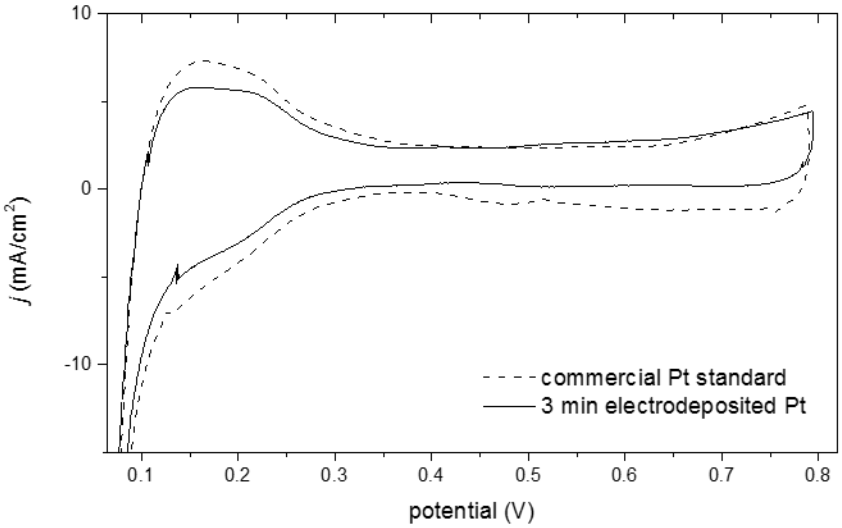

Figure S7. The CV profiles for membrane electrode assemblies (MEAs) prepared from a commercially prepared standard catalyst of Pt nanoparticles (NPs) and ionomer. The mesoporous Pt sample was prepared by 3 min of Pt electrodeposition on top of a film prepared from a mixture of carbon particles and ionomer. The CV profiles were obtained between 0.5 and 0.8 V with a scan rate of 100 mV/s, after completing an initial activation of the catalyst using 200 consecutive CV polarizations.[4]

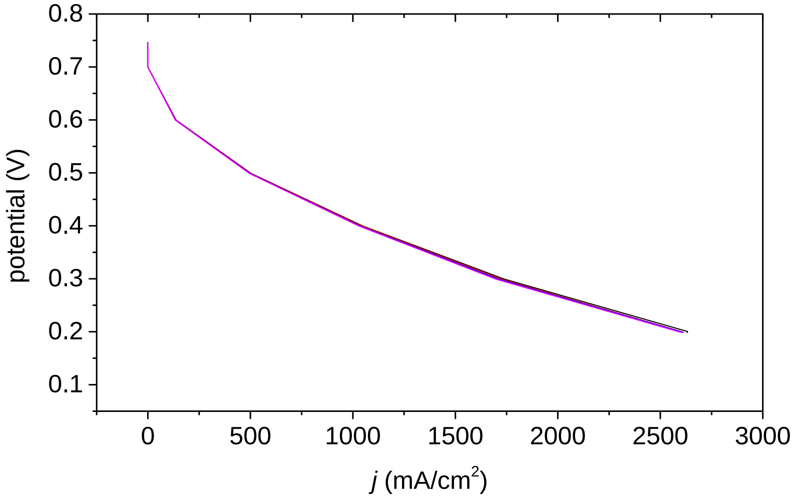

Figure S8. Linear scan voltammetry profiles of the processes used to condition the MEAs prepared with a cathode composed of electrodeposited, mesoporous Pt on a catalyst layer support of carbon and ionomer. A total of 5 profiles are shown with less than 1 mV of variation between each of the successive scans.

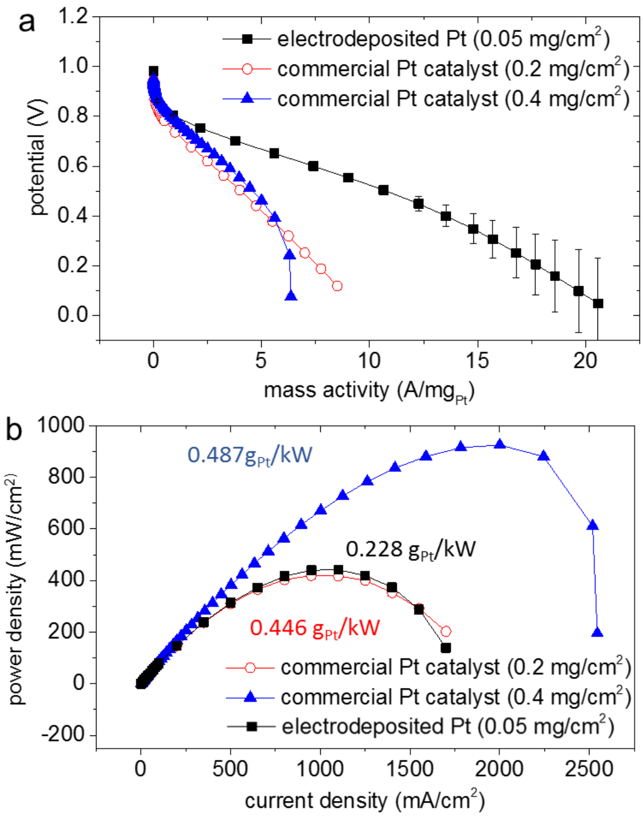

Figure S9. Proton exchange membrane fuel cell (PEMFC) performance using cathodes prepared from the electrodeposited mesoporous Pt in comparison to cathodes prepared from standard Pt nanoparticle based catalysts. (a) Average PEMFC polarization curves plotted as a function of cathode Pt mass activity for the mesoporous Pt (n = 3 samples) and standard commercial catalysts prepared at two different Pt loadings. The respective cathode Pt loadings are included in the legend. (b) Power curves plotted as a function of current density (current versus MEA geometric surface area) for cathodes prepared with mesoporous Pt or commercial Pt nanoparticle based catalysts. The Pt nanoparticle based catalysts were prepared with loadings of 0.4 mg_{Pt}/cm^2 or 0.2 mg_{Pt}/cm^2 as noted in the legend.

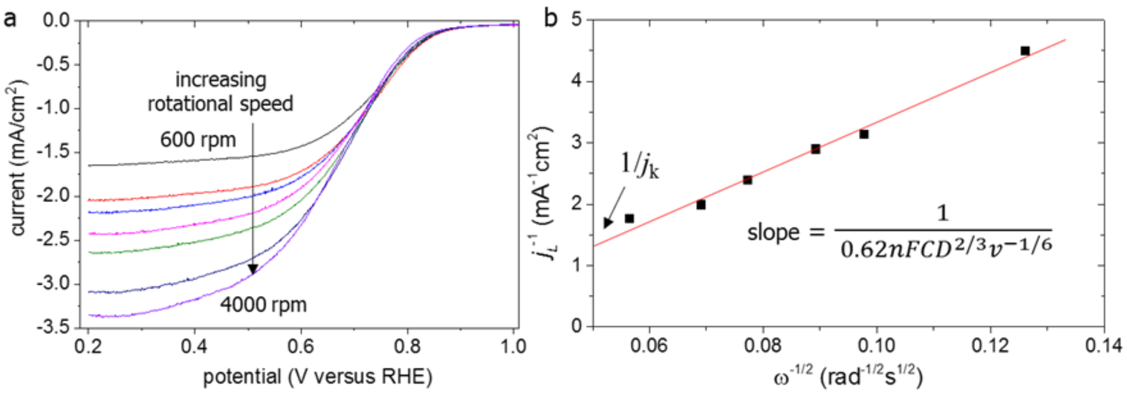

Figure S10. The ORR experimental results of a mesoporous Pt catalyst: (a) LSV plots obtained at different electrode rotational speeds; and (b) a Koutecký-Levich plot obtained by taking the limiting current at 0.2 V at different rotational speeds. The variables in the Koutecký-Levich equation were defined as: n = number of electrode participated in the reaction; F = Faraday's constant; C = oxygen concentration in the analyte; D = diffusion coefficient of oxygen in the analyte; and *v* = rotational speed of the disc electrode.

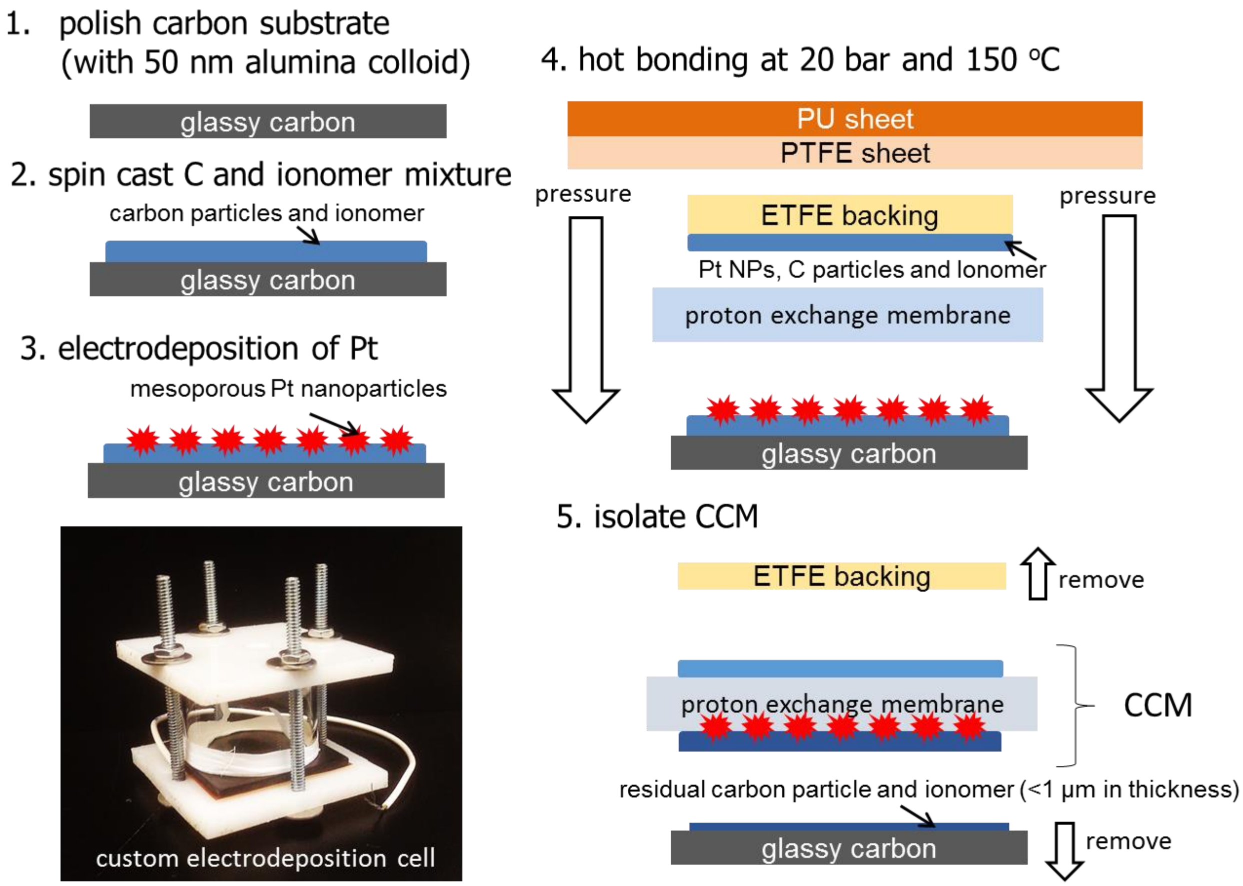

Figure S11. Schematic diagram of the preparation of mesoporous Pt by electrodeposition and its transfer onto proton exchange membranes for the evaluation of these assemblies as custom cathode catalysts layers (CCLs) in PEMFCs. Bottom left picture: the custom electrochemical cell used to prepare the mesoporous Pt on a film of C particles and ionomer coated onto a polished glassy carbon plate. Abbreviations: PU = polyurethane; PTFE = polytetrafluoroethylene [IUPAC: poly(1,1,2,2-tetrafluoroethylene)]; ETFE = ethylene tetrafluoroethylene [IUPAC: poly(1,1,2,2-tetrafluorobutane-1,4-diyl)]; and CCM = catalyst coated membrane.

References

1. Ferreira, P. J. *et al*. Instability of Pt/C Electrocatalysts in Proton Exchange Membrane Fuel Cells. *J. Electrochem. Soc.* 152, A2256 (2005).

2. Tajabadi, M. T. *et al*. Electrodeposition of Flower-Like Platinum on Electrophoretically Grown Nitrogen-Doped Graphene as a Highly Sensitive Electrochemical Non-Enzymatic Biosensor for Hydrogen Peroxide Detection. *Appl. Surf. Sci.* 386, 418–426 (2016).

3. Latsuzbaia, R., Negro, E. & Koper, G. Synthesis, Stabilization and Activation of Pt Nanoparticles for PEMFC Applications. *Fuel Cells* 15, 628–638 (2015).

4. Narayan, C., Purushothaman, S., Doany, F. & Deutsch, A. Thin-film Transfer Process for Low-cost MCM-D Fabrication. *IEEE Trans. Components Packag. Manuf. Technol. Part B-Advanced Packag.* 18, 42–46 (1995).

5. Garland, N., Benjamin, T. & Kopasz, J. DOE Fuel Cell Program: Durability Technical Targets and Testing Protocols. *ECS Transactions* 11, 923–931 (2007).

6. Kongkanand, A. & Mathias, M. F. The Priority and Challenge of High-Power Performance of Low-Platinum Proton-Exchange Membrane Fuel Cells. *J. Phys. Chem. Lett.* 7, 1127–1137 (2016).

7. Ustarroz, J. *et al.* The Role of Nanocluster Aggregation, Coalescence, and Recrystallization in the Electrochemical Deposition of Platinum Nanostructures. *Chem. Mater.* 26, 2396–2406 (2014).
